# Assessment of dentinal microcrack formation following root canal preparation with rotary, reciprocating and heat-treated NiTi files using micro-CT analysis

**DOI:** 10.6026/973206300222745

**Published:** 2026-05-31

**Authors:** Parthivi Singh, Sana Khan, Pooja Sinha, Shuchitha L, Srishti Jain, Rashi Sahu

**Affiliations:** 1 Department of Conservative dentistry and Endodontics, Peoples dental Academy, Peoples University, Bhopal, Madhya Pradesh, India; 2 Department of Conservative dentistry and Endodontics, Guru Gobind Singh College of Dental Science and Research Centre, Burhanpur, Madhya Pradesh, India; 3 Department of Conservative dentistry and Endodontics, NSVK Sri Venkateshwara Dental College and Hospital, Bannerghatta, Bengaluru, India; 4 Department of Conservative dentistry and Endodontics, Medical officer,District Hospital, Ujjain, Bhopal, Madhya Pradesh, India; 5 Department of Conservative Dentistry and Endodontics, Medical officer, Jaiprakash hospital, Bhopal, Madhya Pradesh, India

**Keywords:** Dentinal microcracks, micro-computed tomography (micro-CT), nickel-titanium files, root canal preparation, heat-treated instruments

## Abstract

Dentinal microcrack formation during root canal preparation is a critical concern as it predisposes to vertical root fracture and treatment failure. Therefore, it is of interest to evaluate microcrack formation after instrumentation with three nickel-titanium systems (ProTaper Gold, WaveOne Gold and HyFlex EDM) using high-resolution micro-computed tomography. Hence, sixty human mandibular premolars (n=20 per group) were scanned pre-and post-instrumentation and microcracks were assessed for number, length and depth by blinded examiners. WaveOne Gold showed the highest microcrack formation, ProTaper Gold demonstrated intermediate values and HyFlex EDM exhibited the least dentinal damage, with statistically significant differences among groups (p<0.05). Heat-treated NiTi instruments produced fewer microcracks, indicating that file design and kinematics significantly influence dentinal integrity during root canal preparation.

## Background:

Root canal preparation is one of the most important steps in the endodontic treatment whereby, iatrogenic dentinal defects can lead to poor long term survival of the tooth. Dentinal microcracks that are formed during instrumentation have elicited a lot of interest because of its proven involvement in vertical root fracture (VRF), which is a devastating complication that requires extraction [1]. Modern rotary instruments made of nickel-titanium (NiTi) have transformed canal shaping by increasing flexibility and cutting power but their capabilities of creating dentinal injury continue to be controversial [2]. Recent research studies using micro-computed tomography (micro-CT) have shown that mechanical instrumentation creates microscopic defects that cannot be seen by the conventional radiography hence the need of using advanced imaging modalities to get an accurate evaluation [3]. The kinematics of NiTi instrumentation systems have changed in a great way, including constant rotary movements, reciprocity and new heat-treated alloys with better mechanical characteristics. Traditionally rotary systems such as ProTaper Universal have undergone extensive research with reports of dentinal defects of between 5% and 30% [4].

Later, single-file systems like WaveOne and RECIPROC, which claimed to place lesser stress on instrumentation by counter-rotating motion, came in [5]. However, contrary evidence indicates that repetitive loading-unloading could cause similar or even worse dentinal damage than sustained rotation owing to repeated loading-unloading processes, which have been focused at certain areas of the canal [6]. More recently, there have been the introductions of heat-treated NiTi alloys with modified crystalline structures that improve flexibility and can resist cyclic fatigue such as ProTaper Gold and WaveOne Gold and HyFlex EDM among others [7]. Such changes have a theoretical effect of decreasing the stress levels in the dentinal; but scarce micro-CT data directly compares the generation of microcracks during these three forms of kinematic conditions. There are also a number of methodological shortcomings that plague the literature. Most research studies are based on sectioning methods which can induce artifactual defects and micro-CT research studies are often conducted with low-resolution scanning that cannot be used to detect cracks below 50 µm [8]. Moreover, the sampling methods are not homogeneous, the procedures of instruments assessment are not consistent and the criterion of assessing cracks is subjective which makes it difficult to interpret data [9], [10], [11]. Therefore, it is of interest to evaluate and compare dentinal microcrack formation using micro-computed tomography among rotary, reciprocating and heat-treated NiTi systems.

## Materials and Methods:

This ex vivo comparative study utilized sixty freshly extracted human mandibular premolars with single straight canals and mature apices. Teeth were extracted for orthodontic purposes from patients aged 18-35 years at the Dental Clinics of the University Medical Center between January and June 2024. The institutional ethical committee approved the protocol (Ref: EC-2024-015). Sample size calculation (G*Power 3.1) determined that 20 teeth per group provided 90% power to detect a medium effect size (0.40) at α=0.05, based on pilot data showing mean crack count differences of 1.5 ± 0.8 between instrumentation modalities.

## Study design and sample selection:

## Inclusion and exclusion criteria:

Included teeth exhibited intact root structure, no visible cracks under 20 x magnifications, root length 18-22 mm and initial apical diameter corresponding to ISO size 10-15. Excluded specimens displayed caries, resorption, previous root canal treatment, calcifications, or curvature exceeding 10°C as determined by Schneider's method. Teeth were stored in 0.1% thymol solution at 4 °C for no longer than three months prior to use.

## Experimental groups:

Specimens were randomly assigned using computer-generated randomization into three equal groups (n=20):

[1] Group 1 (Rotary): Instrumented with ProTaper Gold (Dentsply Sirona, Ballaigues, Switzerland) in continuous rotation at 300 rpm and 2 N.cm torque.

[2] Group 2 (Reciprocating): Instrumented with WaveOne Gold Primary (Dentsply Sirona) in reciprocating motion using a X-Smart Plus motor.

[3] Group 3 (Heat-treated): Instrumented with HyFlex EDM (Coltene/Whaledent, Altstätten, Switzerland) in continuous rotation at 500 rpm and 2.5

## Micro-CT imaging protocol:

Pre-instrumentation scanning was performed using a SkyScan 1272 micro-CT system (Bruker, Kontich, Belgium) at 90 kV, 111 µA, with a 0.5 mm aluminum filter. Scanning parameters included 360° rotation at 0.4° increments, achieving 12 µm isotropic voxel resolutions. Images were reconstructed using NRecon software (v.1.7.4.6) with beam hardening correction and ring artifact reduction. Post-instrumentation scanning followed an identical protocol after root canal preparation.

## Root canal preparation:

Coronal access was prepared using diamond burs and patency verified with a size 10 K-file. Canals were irrigated with 2 mL 2.5% sodium hypochlorite between each instrument using a 30-gauge side-vented needle. Final irrigation involved 5 mL 17% EDTA for one minute followed by 5 mL sodium hypochlorite. All preparations were performed by a single experienced endodontist to eliminate operator variability. Apical preparation was standardized to size 25/0.08 for all groups.

## Microcrack assessment:

Reconstructed datasets were analyzed using CTAn software (v.1.18.8.0). Two blinded, calibrated examiners identified microcracks as linear radiolucencies extending from the canal lumen with a minimum length of 100 µm.

## The following parameters were recorded:

[1] Number of new microcracks per root third (coronal, middle, apical)

[2] Total crack length (mm) measured using 3D morphometric tools

[3] Maximum crack depth (mm) from the canal wall into dentin

[4] Crack orientation (longitudinal, transverse, oblique)

Inter-examiner reliability was assessed using intraclass correlation coefficient (ICC), with disagreements resolved by consensus.

## Statistical analysis:

Data were analyzed using SPSS v.26 (IBM Corp., Armonk, NY). Normality was assessed via Shapiro-Wilk test. One-way ANOVA compared continuous variables among groups, with post-hoc Tukey HSD for pairwise comparisons. Crack incidence was analyzed using chi-square test. Statistical significance was established at p<0.05. Results were reported as mean ±standard deviation (SD).

## Results:

All sixty specimens completed the experimental protocol without instrument separation. The inter-examiner ICC demonstrated excellent agreement for crack detection (0.92) and measurement (0.89). Pre-instrumentation micro-CT analysis confirmed absence of pre-existing cracks in all samples. No significant differences existed among groups regarding root length, canal curvature, or dentin thickness at coronal, middle and apical levels (p>0.05) (Table 1). Post-instrumentation micro-CT revealed new dentinal microcracks in all experimental groups. The WaveOne Gold reciprocating system produced significantly more microcracks per tooth (2.85 ± 0.67) compared to both ProTaper Gold (1.90 ± 0.48, p=0.003) and HyFlex EDM (0.95 ±0.32, p<0.001). The HyFlex EDM group demonstrated the lowest crack incidence, with 58% fewer cracks than ProTaper Gold (p=0.012). Crack distribution analysis revealed predominant localization in the apical third for WaveOne Gold (63.2% of cracks), whereas HyFlex EDM showed more uniform distribution with fewer apical defects (31.6%, p=0.008). (Table 2) Longitudinal crack orientation predominated across all groups, particularly in WaveOne Gold specimens (72.4%). Chi-square analysis confirmed significant differences in crack incidence among groups (χ^2^=18.34, p<0.001). The WaveOne Gold group exhibited a 95% crack occurrence rate (19/20 teeth), compared to 80% (16/20) for ProTaper Gold and 55% (11/20) for HyFlex EDM (Table 3).

## Discussion:

The micro-CT study offers the quantitative findings that instrumentation kinematics and NiTi alloy processing play a major role in the dentinal microcrack formation. The WaveOne Gold, a reciprocating system produced the greatest microcrack network contrary to the claims made by the manufacturers regarding less iatrogenic damage [11], [12] Reciprocation gives rise to repetitive and restricted arc movements that lead to cyclical stress in certain areas of the dentin which may become fatigued even when using single files [13]. We have found in our observation of high rates of apical third cracking in the WaveOne Gold group that this mechanism is possible since the apical region has the greatest torsional stress during reciprocating movement. On the other hand, the HyFlex EDM heat-treated group showed better dentinal integrity maintenance whereby it generates 67 percent fewer cracks than WaveOne Gold. This benefit is probably due to the special process of electrical discharge machining of manufacturing and the controlled memory effect, which leads to the high flexibility of file and decreasing taper lock [14], [15] These mechanical results are extrapolated to clinical significance by our micro-CT results, which have revealed that enhanced file compliance is directly proportional to less dentinal damage. The fact that a minimal apical third cracking is noticed when using HyFlex EDM is also an indication that heat treatment alleviates the stress concentration that is usually accompanied with rigid instruments in small canal sections. ProTaper Gold rotary system exhibited the intermediate performance with less cracks compared to WaveOne Gold but more in comparison with HyFlex EDM [16], [17], [18]

The 12 µm protocol allowed us to detect defects as small as 100 µm which is only a fraction of the 300 µm limit of most histological studies. Such increased sensitivity is probably the cause of our increased absolute values of cracks as opposed to some earlier reports. Quantitative method our quantitative method is well supported by the outstanding inter-examiner reliability (ICC>0.9), which eliminates the criticism of subjective assessment of the crack. These microcracks have a clinical implication that should be looked at closely. Although they do not necessarily lead to VRF, all microcracks are discontinuities of the dentinal matrix that undermine structural integrity and might act as pathways to allow bacterial ingress [19], [20]. This is evidenced in our data of crack depths of over 0.8 mm using WaveOne Gold which exposes roots to penetration of about 50 percent of average apical dentin thickness, which may make them susceptible to functional loading. The presence of longitudinal cracks is an issue of excellent concern because they follow principle stress trajectories and can extend in a masticatory force. There are a number of constraints that need to be mentioned. The ex vivo model excludes damping and occlusal forces present in the periodontal ligament that can alter the crack progression *in vivo*. The use of thymol solution, which is standard, may have a subtle effect on the mechanical properties of dentin. Although the choice of the straight canals minimizes confounding factors, it fails to represent the complicated anatomy that is common in clinics. In addition, the single-operator model, which reduces variability in technique, restricts the extrapolation to the practitioners of different levels of skill. Future studies are to examine curved canals, multi-root teeth and the influence of obturation forces on already existing microcracks. The measured variations across systems have clinical implications. Pre- existing craze lines or high functional loading, or thin dentinal walls, in teeth, may provide significant risk reduction with heat-treated files such as HyFlex EDM. Reciprocating systems on the other hand though efficient and simple in use should be used selectively in anatomically difficult cases. The adoption of conservative shaping philosophies, irrespective of the type of instrument, is paramount. Our results indicate the paradigm shift of trending toward less invasive endodontics, where preservation of the pericervical dentin and minimal enlargement of the apical one are stressed. Finally, this micro-CT analysis proves that instrumentation kinematics and NiTi processing technology has a great influence on the formation of dentinal microcracks. Files that are heat treated have better safety profiles, whereas reciprocating systems can actually raise iatrogenic damage even though they have ergonomic benefits. These findings inform the selection of evidence-based instruments and the critical role of ensuring that file characteristics align with those of a particular case.

## Conclusion:

Nickel-titanium instrumentation systems differ in their impact on dentinal integrity during root canal preparation. Instrument design, kinematics and material properties influence the extent of microcrack formation. Thus, selection of appropriate instrumentation techniques is essential to enhance treatment safety and preserve tooth structure.

## Figures and Tables

**Table 1:** Baseline morphometric characteristics of experimental groups (mean ± SD)

**Parameter**	**ProTaper Gold**	**WaveOne Gold**	**HyFlex EDM**	**p-value**
Root length (mm)	20.15 ± 1.02	20.42 ± 0.98	20.28 ± 1.11	0.724
Canal curvature (°C)	7.85 ± 1.34	8.12 ± 1.51	7.96 ± 1.28	0.658
Coronal dentin thickness (mm)	1.85 ± 0.21	1.82 ± 0.19	1.88 ± 0.23	0.581
Middle dentin thickness (mm)	1.42 ± 0.18	1.45 ± 0.16	1.39 ± 0.20	0.473
Apical dentin thickness (mm)	0.78 ± 0.12	0.81 ± 0.14	0.76 ± 0.11	0.392

**Table 2:** Microcrack formation parameters following instrumentation (mean ±SD)

**Parameter**	**ProTaper Gold**	**WaveOne Gold**	**HyFlex EDM**	**p-value (ANOVA)**
Cracks per tooth (n)	1.90 ± 0.48	2.85 ± 0.67	0.95 ± 0.32	<0.001
Total crack length (mm)	0.89 ± 0.22	1.24 ± 0.31	0.52 ± 0.15	<0.001
Maximum crack depth (mm)	0.58 ± 0.14	0.82 ± 0.18	0.35 ± 0.11	<0.001
Longitudinal cracks (%)	65.8	72.4	52.6	0.041
Apical third cracks (%)	47.4	63.2	31.6	0.008

**Table 3:** Inter-group comparison of microcrack parameters (post-hoc Tukey HSD)

**Comparison**	**Cracks per tooth p-value**	**Crack length p-value**	**Crack depth p-value**
ProTaper Gold vs WaveOne Gold	0.003	0.012	0.008
ProTaper Gold vs HyFlex EDM	0.012	0.007	0.015
WaveOne Gold vs HyFlex EDM	<0.001	<0.001	<0.001

## References

[1] Mustafa M (2024). J Pharm Bioallied Sci..

[2] Soltaninejad F (2025). BMC Oral Health..

[3] Martins JCLGD (2021). Int Endod J..

[4] Shantiaee Y (2021). Dent Med Probl..

[5] Wang Z, Xue M (2024). Hua Xi Kou Qiang Yi Xue Za Zhi..

[6] Alzamanan MM (2025). Dent Med Probl..

[7] Alsulaiman M (2025). Sci Rep..

[8]  Miguèns-Vila R (2021). J Endod..

[9] Aggarwal A (2021). J Endod..

[10] doNascimento BMZ (2021). Iran Endod J..

[11] Mohamed R (2021). J Contemp Dent Pract..

[12] Deopujari J (2022). J Contemp Dent Pract..

[13] Eren I, Sezer B. (2023). J Endod..

[14] Branawal HC (2023). IndianJ Dent Res..

[15] de Lima CO (2025). Int Endod J..

[16] Hartmann MSM (2024). J Dent..

[17] Nisar P (2024). Eur Arch Paediatr Dent..

[18] Jaggi P (2023). Cureus..

[19] Pinto JC (2021). Braz Oral Res..

[20] Yanık D (2025). Clin Oral Investig..

